# Znf179 induces differentiation and growth arrest of human primary glioblastoma multiforme in a p53-dependent cell cycle pathway

**DOI:** 10.1038/s41598-017-05305-0

**Published:** 2017-07-06

**Authors:** Kuen-Haur Lee, Chi-Long Chen, Yi-Chao Lee, Tzu-Jen Kao, Kai-Yun Chen, Chih-Yeu Fang, Wen-Chang Chang, Yung-Hsaio Chiang, Chi-Chen Huang

**Affiliations:** 1 Graduate Institute of Cancer Biology and Drug Discovery, College of Medical Science and Technology, Taipei Medical University, Taipei, Taiwan; 2 Department of Pathology, Taipei Medical University Hospital, Taipei, Taiwan; 3 Department of Pathology, School of Medicine, College of Medicine, Taipei Medical University, Taipei, Taiwan; 4 Graduate Institute of Neural Regenerative Medicine, College of Medical Science and Technology, Taipei Medical University, Taipei, Taiwan; 5 Department of Pathology, Wan Fang Hospital, Taipei Medical University, Taipei, Taiwan; 6 Graduate Institute of Medical Sciences, College of Medicine, Taipei Medical University, Taipei, Taiwan

## Abstract

Malignant glioblastoma multiforme (GBM) is an aggressive brain tumor with strong local invasive growth and a poor prognosis. One probable way to manipulate GBM cells toward a less invasive status is to reprogram the most malignant GBM cells to a more differentiated and less oncogenic phenotype. Herein, we identified a novel role of a RING finger protein Znf179 in gliomagenesis. Znf179 overexpression induced differentiation of primary GBM cells, which were accompanied with elevated glial fibrillary acidic protein (GFAP) expression through up-regulating several cell-cycle-related factors, p53, p21, and p27, and allowed the cell-cycle arrest in the G_0_/G_1_ phase. In addition, Znf179 was highly correlated with the prognosis and survival rates of glioma patients. The expression levels of Znf179 was relatively lower in glioma patients compared to normal people, and glioma patients with lower expression levels of *Znf179* mRNA had poorer prognosis and lower survival rates. In conclusion, we provide novel insight that Znf179 can reprogram GBM cells into a more-differentiated phenotype and prevent the progression of gliomas to a more-malignant state through p53-mediated cell-cycle signaling pathways. Understanding the molecular mechanism of Znf179 in gliomagenesis could help predict prognostic consequences, and targeting Znf179 could be a potential biomarker for glioma progression.

## Introduction

Gliomas, derived from glial cells, are the most common lethal primary brain tumor in adults due to their radio- and chemoresistance^1,2^. Genetic mutations and deletions of tumor-suppressor genes or cell-cycle regulators are thought to be causes of gliomas^3^. According to the 2007 World Health Organization (WHO) classification, gliomas are graded according to the extent of anaplasia (“de-differentiation”), which implies biological aggressiveness in microscopic features, such as mitotic activity, tumor necrosis, and angiogenesis^4^. Following this scheme, more-malignant tumors resemble less-differentiated precursor cells. Patients with the most malignant human brain tumor, the highly invasive and proliferative glioblastoma multiforme (GBM), have very poor prognosis with averaged <12-month survival period from the time of diagnosis. It was recently accepted that undifferentiated tumor cells, called cancer stem cells (CSCs), play pivotal roles in the initiation and progression of cancers in various tissues^5^. CSCs comprise only a small portion of a tumor, and each single cell can further develop into a new portion of a tumor. Recent studies suggested that the uncontrolled renewal potential of CSCs in GBM cells might be the driving force behind recurrent tumorigenesis and the reason for the failure of conventional cancer therapies^6^. Recurrent tumors are more malignant, fast spreading, and resistant to radiotherapy and previously used drugs, leading to the relapse of GBM with worse prognosis. Delayed tumor recurrence from the residual CSC pool within GBM cells by driving CSCs into differentiation is an imperative antitumor action of GBM. Therefore, identifying key factors that are able to reprogram malignant GBM cells to a more-differentiated, less-oncogenic phenotype could highly extend the probability of manipulating the GBM cells toward a less-aggressive circumstances^7–9^.

A RING finger family protein, Znf179, the gene of which is located within the Smith-Magenis syndrome region on chromosome 17, has been recently demonstrated to play a critical role in neuronal differentiation^10–12^. During brain development, Znf179 is predominantly expressed in the brain region including the cerebral cortex, hippocampus, lateral amygdaloidal nucleus, ventromedial hypothalamus, and cerebellum^11,12^. Its expression gradually increases during embryogenesis in the developing brain and reaches the highest level in the adult stage. Previously, we found that knockdown of Znf179 resulted in decreased expressions of the negative cell-cycle regulators, p35 and p27, leading to reprogramming of the cell cycle and impaired neuronal differentiation^10^. During normal brain development, deregulation of gliogenesis that interferes with differentiation of neural stem cells (NSCs) into astrocytes might contribute to glioma formation^13^. Genetic alterations in signal transduction routes and processes induced by growth factors which control cell cycle progression, such as receptor tyrosine kinases (RTKs)/RAS/phosphatidylinositol 3-kinase (PI-3K), the p53 tumor suppressor pathway, and retinoblastoma (RB) protein signaling pathways, are found in majority of GBM tumors. Malfunction of these cell-cycle regulators can enhance cell proliferation and survival while allowing GBM tumor cells to escape cell-cycle checkpoints^14,15^. Even though the *Znf179* gene was cloned more than 20 years ago, its function still has not been thoroughly elucidated. Our previous studies have shown that Znf179 is a potent key factor inducing neuronal differentiation through attenuating cell-cycle regulation. The biological roles of Znf179 in the process of glioma formation (gliomagenesis), however, have never been discussed. It thus draws our interest to investigate whether Znf179 can reprogram malignant GBM cells to a more-differentiated, less-aggressive circumstance, and the cell cycle-related signal cascades Znf179 involved during gliomagenesis. Exploring the role of Znf179 in glioma can provide essential information about novel targets for glioma therapies.

Many studies indicated that histological anaplasia, mitotic activity, tumor necrosis, and angiogenesis as important prognostic factors for grade classification of gliomas. However, these clinical parameters do not take fully account of observed variations in survival rates or the prognosis of both high- and low-grade tumors^16^. Therefore, additional indicators are required to determine more accurately the prognosis of patients with gliomas. Due to advances studies in microarray and genomic sequencing, more detailed genetic and epigenetic changes in glioma tumors at the genome-wide level have been evaluated^14^. Herein, we used a genomic database to analyze the correlation between Znf179 expression and prognosis in glioma patients, and found that *Znf179* messenger (m)RNA and protein expression levels were relatively lower in glioblastoma tissues compared to normal tissues. In addition, decreased expression of *Znf179* mRNA in glioma patients was accompanied by poorer prognostic outcomes and lower survival rates. In primary GBM cells, overexpression of Znf179 can induce significant differentiation of GBM cells accompanied by increased GFAP expression. Moreover, overexpression of Znf179 caused arrest of the cell cycle in the G_0_/G_1_ phase through up-regulation of p53, p21, and p27 cell-cycle signaling cascades in primary GBM cells. In combined, our findings suggest that Znf179 can attenuate proliferation rates of GBM cells and reprogram GBM cells into a more-differentiated phenotype through p53-p21-p27 cell-cycle signal regulation. Targeting of Znf179’s role in glioma reprogramming may provide insight in novel development of prognostic biomarkers and potential treatment targets for glioma patients.

## Results

### Znf179 induces neuronal differentiation

**Figure 1:**
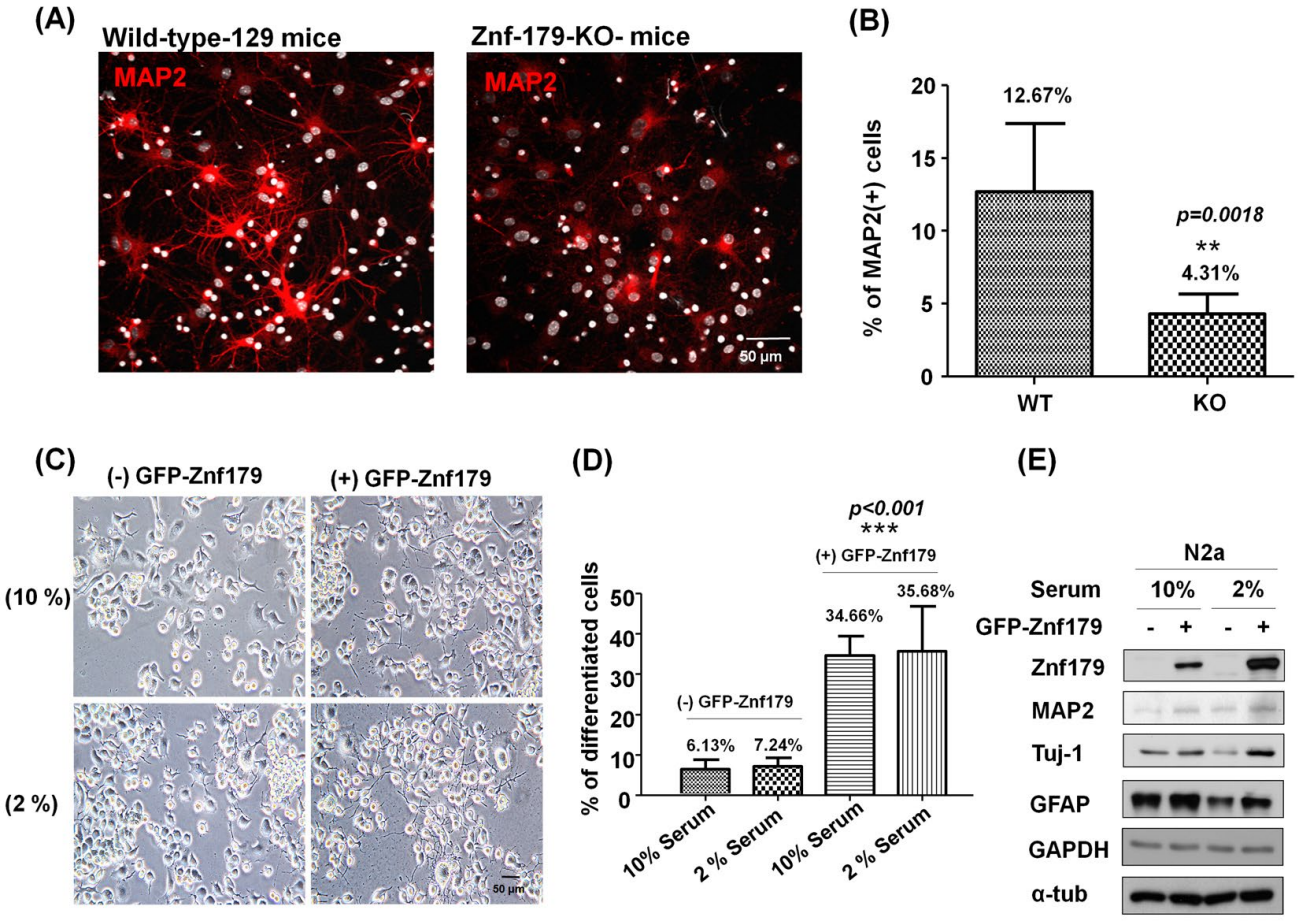
Effect of Znf179 on neuronal differentiation. **(A,B**) Knockdown of Znf179 expression diminished neuronal differentiation and dendritic arborization in primary cerebellar granule cells. Primary culture of cerebellar granule cells from cerebella of wild-type or Znf-179-knockout mice were stained with anti-MAP2 (red) and DAPI (white). The Quantification of the percentage of MAP2(+) cells is shown in the histogram. **p = 0.0018. Scale bar, 50 μm. (*n* > 300, groups were compared using *t*-test, two-tailed *p* value). **(C,D)** N2a cells with and without stable expression of GFP-Znf179 proteins were induced to differentiate in MEM containing 2% serum for 24 h. The overexpression of GFP-Znf179 induced N2a cell differentiation in both 10% and 2% serum-containing medium, while those in 2% serum-containing medium exhibited longer neurite lengths (the lower right panel in 1 C). Significant neurite branching was observed in differentiated N2a cells. Differentiated and total N2a cells were determined in at least five fields of views, and at least 600 cells were counted in total. Quantification of the percentage of differentiated N2a cells is shown in the histogram. ***p < 0.001. Scale bar, 50 μm. (*n* > 600, groups were compared by a one-way ANOVA, two-tailed *p* value). **(E)** Total cell lysates of N2a cells were analyzed by Western blotting using anti-MAP2, anti-Znf179, anti-Tuj-1, anti-GFAP, anti-α-tubulin and anti-GAPDH antibodies. Uncropped images are in Supplementary Figure S2.

Our previous studies demonstrated that Znf179 is a potent neuronal differentiation initiator^10^. To further support this notion, we analyzed the neuronal morphology of N2a cells and primary granule cells from the cerebellum of wild-type and Znf179-knockout mice. Primary culture of cerebellar granule cells from the cerebellum of 7-day-old (P7) wild-type and Znf-179-knockout mice were cultured for 15 days *in vitro* (D﻿.I﻿.V) and stained with anti-MAP2 (red) and DAPI (white). MAP2 is a marker of soma and dendrites, and MAP2(+) cells represent granule cells with significant neurite branching and dendritic arborization. Knockdown of Znf179 expression diminished neuronal differentiation and dendritic arborization of primary cerebellar granule cells, accompanied by decreased expression of the MAP2 neuronal marker (Fig. 1A,B), which is consistent with previously observed results^10^. In contrast, N2a cells stably expressing GFP-Znf179 had differentiated into a neuron-like morphology (Fig. 1C,D), especially following treatment of differentiation medium containing 2% serum, which further confirmed the notion that Znf179 is a potent initiator of neural differentiation^10^. Expression levels of the neuronal markers, Tuj-1 and MAP2, and the glial marker, GFAP, were increased in Znf179-overexpressing N2a cells (Fig. 1E), especially following treatment of differentiation medium containing 2% serum, suggesting that Znf179 plays an important role in neuronal differentiation, including neurogenesis and gliogenesis, during brain development.

### Decreased expression of Znf179 in glioma brain tumors

**Figure 2:**
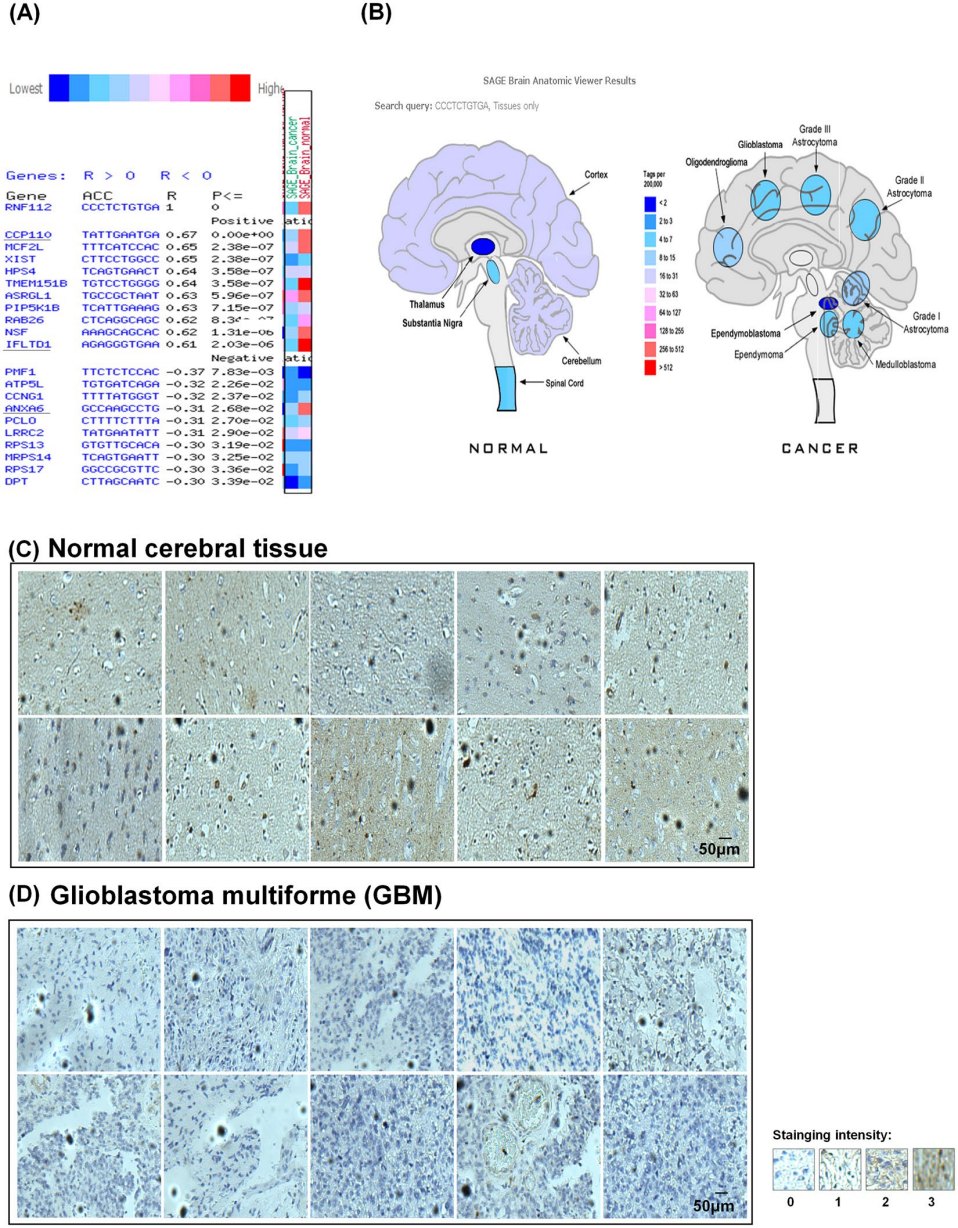
Expression patterns of Znf179 in normal cerebral tissues and astrocytoma tissues. **(A)** Gene expression patterns of *Znf179* (*Rnf112*) in multiple malignancies compared to their normal counterparts in CGAP gene expression databases. In brain cancers, expression levels of *Znf179* (*Rnf112*) genes were lower compared to normal brains. **(B)** The results obtained from the public SAGE brain anatomic viewer (https://cgap.nci.nih.gov/SAGE/AnatomicViewer) showed that most gene expression patterns of *Znf179* were distributed among the brain cortex, and the expression levels of *Znf179* (*Rnf112*) were lower in astrocytomas, compared to normal brain tissues, and were even lower in higher-grade astrocytomas. **(C** and **D)** Brain tissue microarray of normal cerebral (*n* = 27) **(C)** and grade IV glioblastoma multiforme (GBM) tissues (*n* = 42) **(D)** was analyzed by IHC with an anti-Znf179 antibody. Staining intensities from score 0 to 3 are shown. Scale bar: 50 μm.

Deregulation of neural stem cell (NSC) differentiation into astrocytes during normal brain development or gliogenesis might contribute to glioma formation^13^. The involvement of Znf179 in neuronal differentiation during brain development was well demonstrated in our previous results^10^ and is also shown in Fig. 1. However, the biological functions of Znf179 in glioma tumorigenesis have never been discussed. To investigate the functional roles and molecular mechanism of Znf179 in gliomagenesis, we first compared gene expression levels of *Znf179* (*Rnf112*) in normal and cancerous brain tissues from a CGAP gene expression database (National Cancer Institute Cancer Genome Anatomy Project; http://cgap.nci.nih.gov/) and a SAGE brain anatomic viewer (https://cgap.nci.nih.gov/SAGE/AnatomicViewer). Down- or upregulation of *Znf179* (*Rnf112*) was observed in multiple malignancies compared to their normal counterparts (Fig. 2A). Expression levels of *Znf179* (*Rnf112*) genes in cancerous brains were lower than in normal brains (Fig. 2A). Results of the SAGE brain anatomic viewer showed that most gene expression profiles of *Znf179* were distributed among the cortex and cerebellum in normal brains (light-purple area in the left normal brain, Fig. 2B), while expression levels of *Znf179* were lower in glioma tissues (light-blue area in grade I~III astrocytomas and glioblastomas in the right cancerous brain, Fig. 2B) compared to normal brains (light-purple area in the left normal brain, Fig. 2B). In higher-grade astrocytomas, gene expression levels of *Znf179* were even lower (Fig. 2B). To confirm results of the CGAP gene expression database, we then compared relative protein expression levels of Znf179 in normal cerebral and grade IV GBM tissues by IHC. We found that the expression levels of Znf179 were relatively lower in malignant grade IV GBM tissues (*n* = 42) compared to those in normal cerebral tissues (*n* = 27). Znf179 expression was downregulated in representative images of ten malignant grade IV GBM tissues (Fig. 2C,D). In combined, these results suggested that Znf179 expression is involved in repression of gliomagenesis and may play a function role as a tumor suppressor during glioma progression.

### Znf179 overexpression enhances differentiation of GBM cells

**Figure 3:**
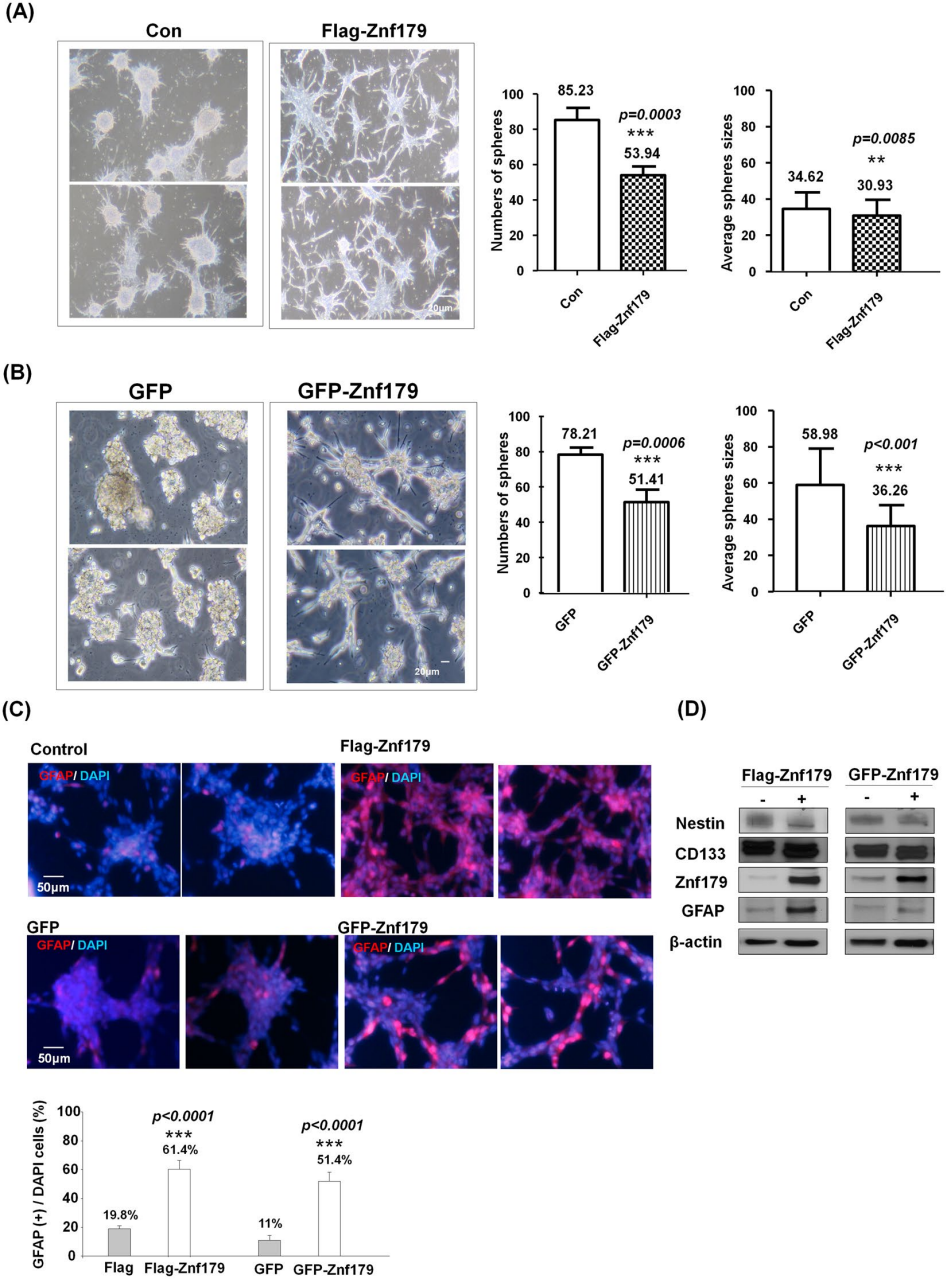
Overexpression of Znf179 in primary glioblastoma multiforme (GBM) cells promotes neuronal differentiation accompanied by the induction of the glial marker, glial fibrillary acidic protein (GFAP). Primary GBM-derived cells stably expressing Flag-Znf179 (**A**) or GFP-Znf179 (**B**) were differentiated in DMEM/F12 medium containing 1% FBS as described in “Materials and Methods”. Average numbers of glioma tumor spheres and sphere sizes of GBM cells were determined in at least three independent experiments. Scale bar: 20 μm. *p < 0.05; **p < 0.01; ***p < 0.001 (groups were compared by *t*-test, two-tailed *p* values). (**C**) Primary GBM-derived cells stably expressing Flag-Znf79 or GFP-Znf179 were differentiated and immunostained with an anti-GFAP antibody. The GFAP(+) differentiated GBM cells with significant long neurite branching were shown in red. Quantification of the percentage of GFAP(+) cells is shown in the histogram. ***p < 0.001 (groups were compared by a *t*-test, two-tailed *p* values). Scale bar: 50 μm. (**D**) Total lysates of these GBM cells were analyzed by Western blotting using anti-Znf179, anti-CD133, anti-nestin, anti-GFAP, and anti-β-actin antibodies. Uncropped images are in Supplementary Figure S3.

Our previous studies demonstrated that knockdown of Znf179 inhibits neuronal and glial differentiation in cerebellar granule cells, whereas overexpression of Znf179 enhances neuronal differentiation (Fig. 1). In the CGAP database analysis and IHC results, gene and protein expression levels of Znf179 were much lower in glioma tissues compared to normal brain tissues, suggesting that Znf179 may play a role as a suppressor to repress the transformation, viability, and malignant progression of gliomas, which promoted us to study the effect of Znf179 on gliomas. Glioma tumor spheres derived from a patient with WHO grade IV GBM were transfected with Flag-tagged or GFP-tagged Znf179 plasmids and selected by G418 to generate a GBM-Znf179 stable cell line that stably expressed Znf179. We then analyzed the population of adherent differentiated glial cells and glioma tumor spheres in these Znf179-transfected GBM cells. GBM cells that formed suspended or attached smooth-edged, bulk-like colonies without significant breaching neurites were identified as glioma tumor spheres, while attached, flat, spiny colonies with long branching neurites were identified as differentiated GBM cells. We found that average numbers of glioma tumor spheres and sphere sizes in primary GBM cells stably expressing Znf179 had decreased, and most of the GBM tumor spheres overexpressing Znf179 had differentiated into adherent glial cells (Fig. 3A,B). Moreover, the glial-specific marker, GFAP, had increased in differentiated primary GBM cells which stably expressed Znf179 (Fig. 3C,D), suggesting that Znf179 can reprogram GBM cells into a more-differentiated phenotype. Neural stem/progenitor cell markers, CD133 and nestin, were equivalent among Znf179-overexpressing or non-overexpressing GBM cells (Fig. 3D). In combined, these results suggested that Znf179 induces the differentiation of GBM-derived cells and reduces the tumor burden, while the cell populations remained in the neural progenitor state are not affected.

### p53-p21-p27 cell cycle signaling pathways are involved in Znf179-induced differentiation of GBM cells

**Figure 4:**
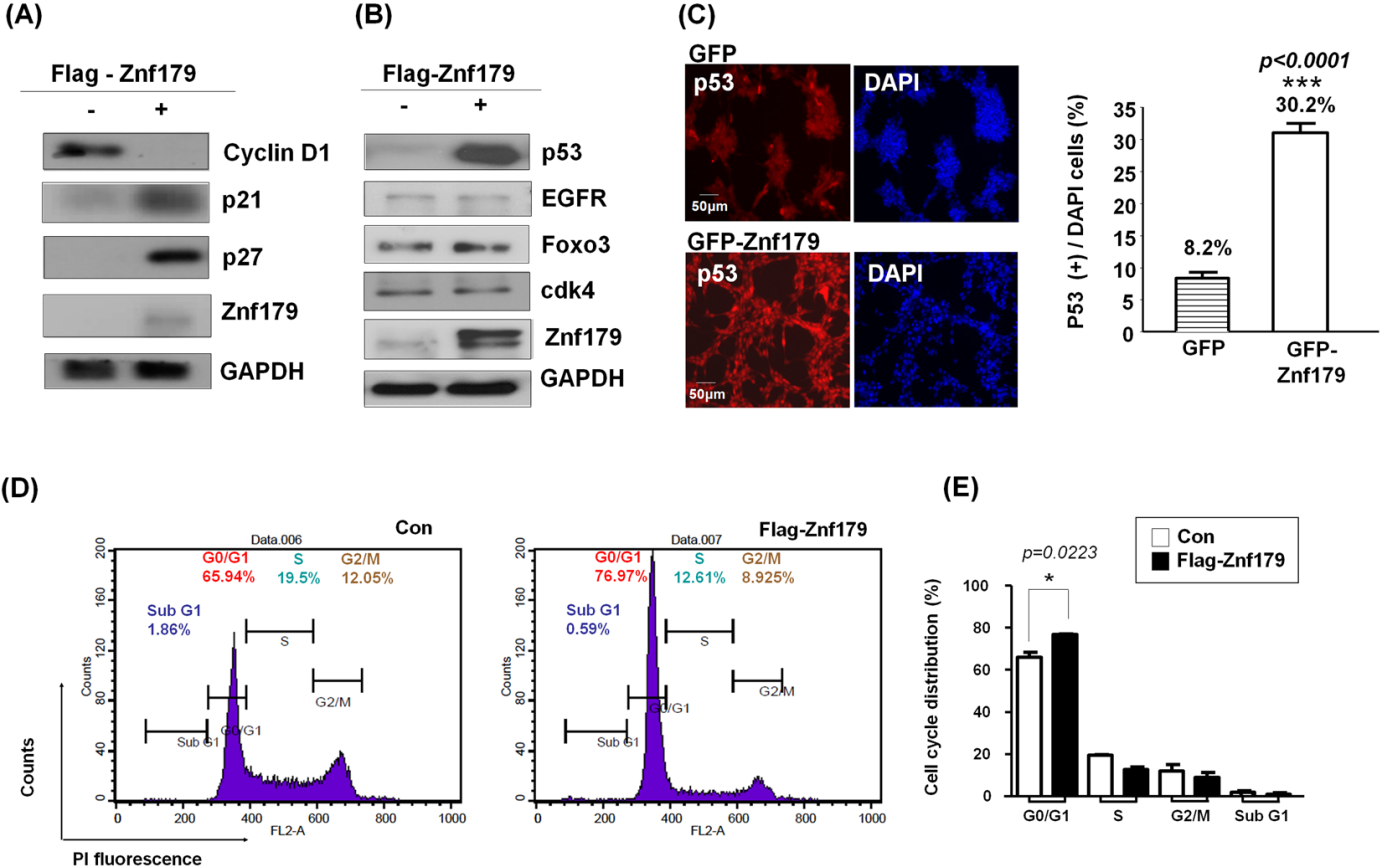
Znf179 arrests glioblastoma multiforme (GBM) cells in the G_0_/G_1_ phase through p53-p21-p27 cell-cycle signaling pathways. (**A** and **B**) Primary GBM-derived cells stably expressing Flag-Znf79 were differentiated in DMEM/F12 medium containing 1% FBS as described in “Materials and Methods”, and total lysates of these GBM cells were analyzed by Western blotting. Uncropped images are in Supplementary Figure S4. (**C**) Primary GBM-derived cells stably expressing GFP-Znf79 were differentiated and immunostained with an anti-p53 antibody. Quantification of the percentage of p53(+) cells is shown in the histogram. ***p < 0.001 (groups were compared by a t-test, two-tailed *p* values). Scale bar: 50 μm. (**D**) Primary GBM-derived cells stably expressing Flag-Znf79 were differentiated, and the cell cycle distribution was detected by flow cytometry. (**E**) Data are presented as the mean ± SEM (*n* = 2, *p = 0.0223, Flag-179-G_0_/G_1_ compared to Flag-G_0_/G_1_ by a *t*-test, two-tailed *p* values).

Three major signal pathways correlated with cell survival, cell cycle regulation, and cell apoptosis were identified as pathological signatures for gliomagenesis, including the RTK/RAS/PI-3K, p53 tumor-suppressor, and RB signaling pathways^14,15^. Therefore, we next analyzed theexpression levels of these cell growth or cell-cycle-related factors involved in pathological gliomagenesis with or without Znf179 overexpression. In primary GBM cells stably expressing Flag-Znf179, we found that expression levels of the G_1_ phase cell cycle regulator, cyclin D1, were downregulated (Fig. 4A). We then determined the expression levels of the cyclin-dependent kinase inhibitors, p21 and p27, and found that both p21 and p27 were upregulated in Znf179-overexpressing GBM cells (Fig. 4A). These results thus indicate that Znf179 induces growth arrest of GBM cells through downregulating cell-cycle regulators. To further understand signal pathways involving Znf179 during gliomagenesis, we also analyzed the expressions of glioma pathological molecular factors, including p53 (a ARF-MDM2-p53-related factor), EGFR and Foxo3 (RTK-PI-3K-related factors), and cdk4 (a INK4a-Rb-related factor) in primary GBM cells stably expressing Flag-Znf179. Expression levels of p53 were higher in Znf179-overexpressing primary GMB cells, whereas EGFR, Foxo3, and cdk4 expression patterns were similar in primary GBM cells with or without Znf179 overexpression (Fig. 4B). We further analyzed expression patterns of p53 in Znf179-overexpressing GBM cells by an immunofluorescent assay, and found that p53 increased when Znf179 was overexpressed in GBM cells (Fig. 4C). Since p53 is a tumor-suppressor protein that is critical for cell-cycle arrest, apoptosis, and DNA repair, we further analyzed the cell-cycle distribution of Znf179-overexpressing GBM cells by flow cytometry, and found that Flag-Znf179-overexpressing GBM cells were mostly arrested in the G_1_/G_0_ phase, compared to the control groups (Fig. 4D,E). In summary, our results demonstrated that Znf179 can arrest GBM cells in the G_0_/G_1_ phase through p53-p21-p27 cell cycle signaling pathways and reprogram GBM cells into a more-differentiated phenotype.

### Glioma patients with higher *Znf179* expression levels have longer survival rates

**Figure 5:**
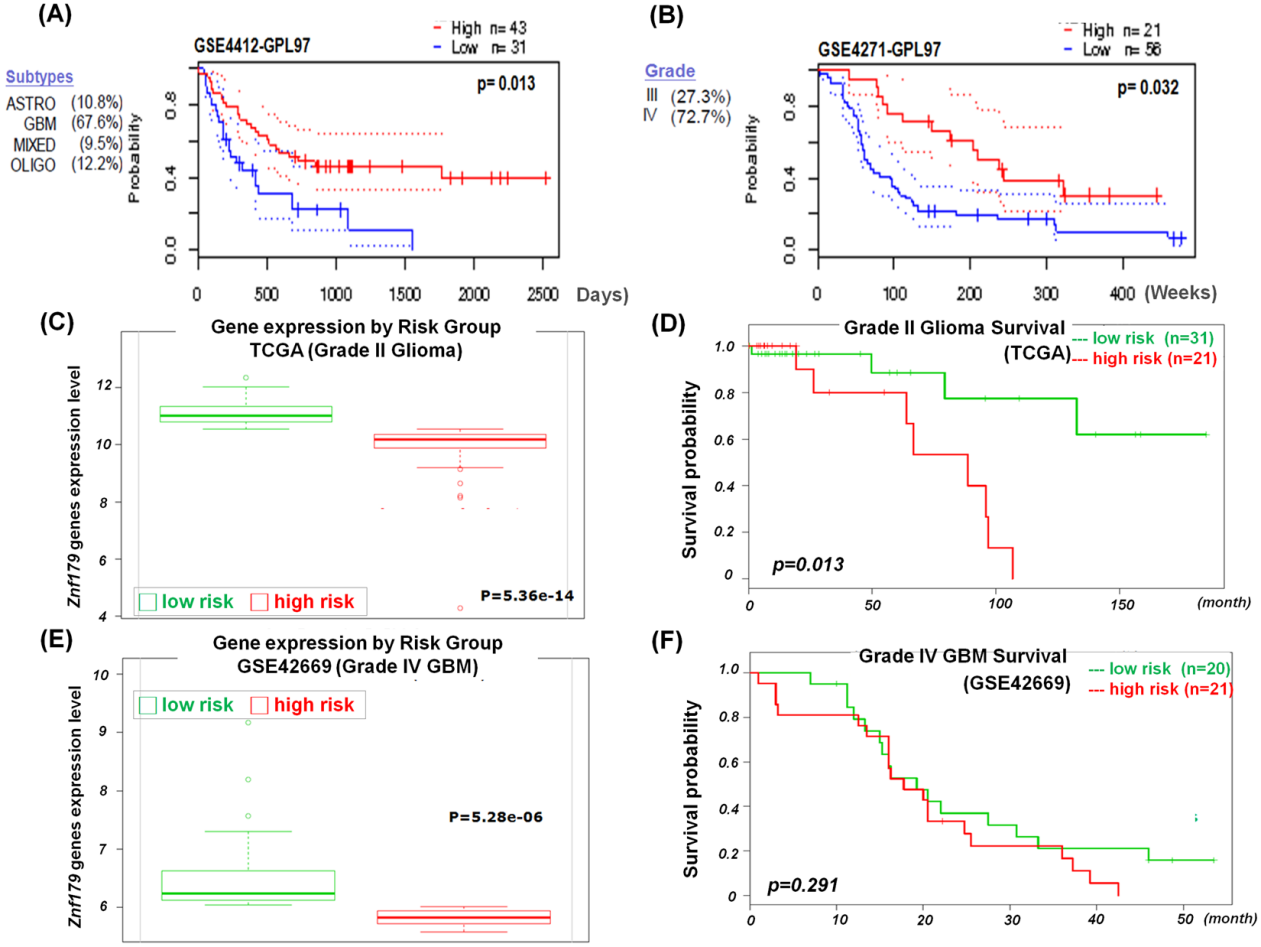
Correlation between *Znf179* expression and the overall survival rates in glioma patients. **(A** and **B)** The survival curve comparing patients with high (red) and low (blue) Znf179 expression was plotted from the PrognoScan database. In the Kaplan-Meier overall survival plot of both glioma databases, patients with higher expression levels of Znf179 had better overall survival rates. **(C** and **D)** Box plots comparing differences in Znf179 gene expression of grade II gliomas (**C**) and grade IV glioblastoma multiforme (GBM) (**F**) between two risk groups using a *t*-test. **(E** and **F)** Accurately predicting survival rates using Kaplan-Meier analyses of patients with different expressions of Znf179 from grade II gliomas (**E**, *n* = 52, *p* = 0.013) and grade IV GBM (**F**, *n* = 42, *p* = 0.291).

The above results indicated the important functions of Znf179 in glioma differentiation. Next, we analyzed the clinical prognostic relevance of Znf179 in glioma cancer patients using publically available GEO expression profiles obtained from the PrognoScan database^17^. In the GSE4412-GPL97 database (*n* = 74) containing 67% malignant grade IV GBM and 32.4% astrocytomas or other glioma patients, those with low *Znf179* expression levels (*n* = 31) were significantly associated with a poor prognosis and survival rates (*p* = 0.013) (Fig. 5A). In another GSE4271-GPL97 database (*n* = 77) containing 27.3% malignant grade III astrocytomas and 72.7% grade IV GBM patients, those with low *Znf179* expression levels (*n* = 56) were significantly associated with a poor prognosis and survival rates (*p* = 0.032) (Fig. 5B). These results thus suggested that expression levels of *Znf179* is highly relevant with glioma patient’s survival rates and is a potential prognostic predictor for glioma patients. To better understand the correlation between *Znf179* gene expression and survival rates in patients with different-grade gliomas, cohorts of 52 low-grade II glioma patients (TCGA) (Fig. 5C,D) and 41 malignant grade IV GBM patients (GSE42669) (Fig. 5E,F**)** were analyzed using a Kaplan-Meier survival analysis. In Fig. 5C, expression levels of *Znf179* were used to stratify grade II glioma patients into two subgroups (low- and high-risk groups): the low-risk group with higher *Znf179* expression levels exhibited longer survival rates (green line in Fig. 5D), whereas the high-risk group with lower *Znf179* expression exhibited shorter survival times (red line in Fig. 5D). In grade II glioma patients, *Znf179* expression shown in two risk groups exhibited significant differences (*p* = 0.013) in clinical outcomes according to the Kaplan-Meier survival analysis (Fig. 5D). However, in grade IV GBM patients, expression levels of *Znf179* in high- and low-risk groups (Fig. 5E) were both much lower than that in grade II glioma patients (Fig. 5C), and there was no significant difference in clinical outcomes (*p* = 0.291) according to the Kaplan-Meier survival analysis (Fig. 5F). These results indicate that the expression level of *Znf179* is a prominent predictor of survival rates of glioma patients. Therefore, we conclude that Znf179 can repress the malignant progression when it is overexpressed in gliomas, and serve as a clinically promising prognostic biomarker and novel molecule target for glioma patients.

## Discussion

In this study, we provided a novel insight that Znf179 can reprogram GBM cells into a more differentiated phenotype and attenuate the progression of glioma to a more malignant state. There are several lines of evidence that support this conclusion which are listed below. First, we found that Znf179 expression in malignant grade IV GBM tissues was downregulated compared to that in normal cerebral tissues. Second, Znf179 delayed cell-cycle and attenuated cell growth in GBM-derived cells. Third, in human GBM cells, Zfn179 regulated expression levels of the tumor suppressor, p53, and its downstream targets of p21 and p27. Forth, Znf179 accelerated the differentiation of GBM cells, which was accompanied by induction of expression of the GFAP glial marker. Collectively, our findings suggest that the p53/p27/p21 signaling pathway is involved in Znd179-induced GBM differentiation via arresting the cell cycle following upregulation of GFAP.

In current study, we demonstrated that Znf179 knockdown in primary cerebellar granule cells diminished neuronal differentiation, dendritic arborization, and decreased expression of the MAP2 neuronal marker (Fig. 1A,B). On the other hand, GFP-Znf179 expressing N2a cells were more susceptible to differentiate along with increased expressions of Tuj-1 neuronal marker and GFAP glial marker (Fig. 1C,D). These results further confirmed that Znf179 is a potent initiator of neural differentiation during brain development, including neurogenesis and gliogenesis, which is consistent with our previous findings^10^.

In this study, we used one human primary GBM cell line derived from a patient with WHO grade IV GBM, and we transfected this GBM cell line with Flag-tagged or GFP-tagged Znf179 plasmids to generate two GBM-Znf179 stable cell lines that stably express Znf179 (Fig. 3). We performed all experiments with these two stable GBM-Znf179 cells lines to analyze the effect of Znf179 on GBM differentiation. In both Flag-tagged and GFP-tagged Znf179-overexpressing GBM cells, average numbers and sizes of glioma tumor spheres were decreased, and numerous GBM sphere cells overexpressing Znf179 had differentiated into adherent glial cells. Coincidently, our quantitative analysis also showed a reduction of the G_1_ phase cell-cycle regulator, cyclin D1, and induction of p53/p27/p21 expression in these two stable GBM-Znf179 cell lines.

In our cell-cycle analytical assays, the sub-G_1_ cell population in GBM-Znf179( + ) cells was relatively lower than those in GBM-Znf179(−) cells, yet the difference was not significant (Fig. 4D,E). These results suggested that Znf179-induced cell differentiation resulted from arresting the cell cycle in the G_0_/G_1_ phase and attenuating proliferation rates, instead of inducing cell apoptosis, which is consistent with our previous findings^10^. Here we also quantified protein expression levels of the neural progenitor/precursor marker, nestin, and performed nestin staining as shown in Supplementary Fig. S1. In the Western blotting and immunostaining assays, effects of Znf179 on expression levels of nestin proteins in GBM cells were similar. In addition, protein expression levels of another neural progenitor/precursor marker, CD133, were also unchanged (Fig. 3D). These results thus suggested that the antitumor effect of Znf179 is through up-regulating differentiation of GBM-derived cells and reducing the tumor burden, but not through depleting the remaining cell population in the neural progenitor state or eradicating resistant cancer-initiating cells.

It is well-known that increased production of reactive oxygen species (ROS) and altered redox status can be observed in cancer cells. The increased ROS levels are able to promote tumor growth and malignancy by promoting DNA damage and genomic instability, which then activate inflammatory responses^3^, stabilize hypoxia-inducible factor-1 and thus reprogram metabolism^18^. Protecting cells against ROS could thus be a potential anti-tumorigenic strategy. Recently, we identified a novel role of Znf179 as a neuroprotector against oxidative stress: Znf179 has been shown to involve in sigma-1 receptor (Sig-1R; a chaperone of endoplasmic reticulum)-mediated antioxidant effect, and overexpression of Zn179 reduces oxidative injury via increasing antioxidant enzyme levels^19^. Therefore, in addition to the promotion of GBM differentiation through arresting cell cycle in G_0_/G_1_ phase, Znf179 could also have other anti-tumor functions through the protection of neuron cells against ROS damage and the attenuation of tumorigenesis through the reduction of oxidative stress signaling pathway. Overall speaking, we demonstrated several antitumor effects of Znf179 which works through 1) arresting GBM cells in the G_0_/G_1_ phase through p53 cell cycle regulation, 2) downregulating cellular proliferation rates, 3) protecting neuron cells against ROS damage and reducing oxidative stress signaling pathways. Other possible means such as mediating cellular apoptosis, depleting the cell populations remained in a neural progenitor state, or eradicating resistant cancer-initiating cells may not be considered as the anti-tumor effects of Znf179.

**Figure 6:**
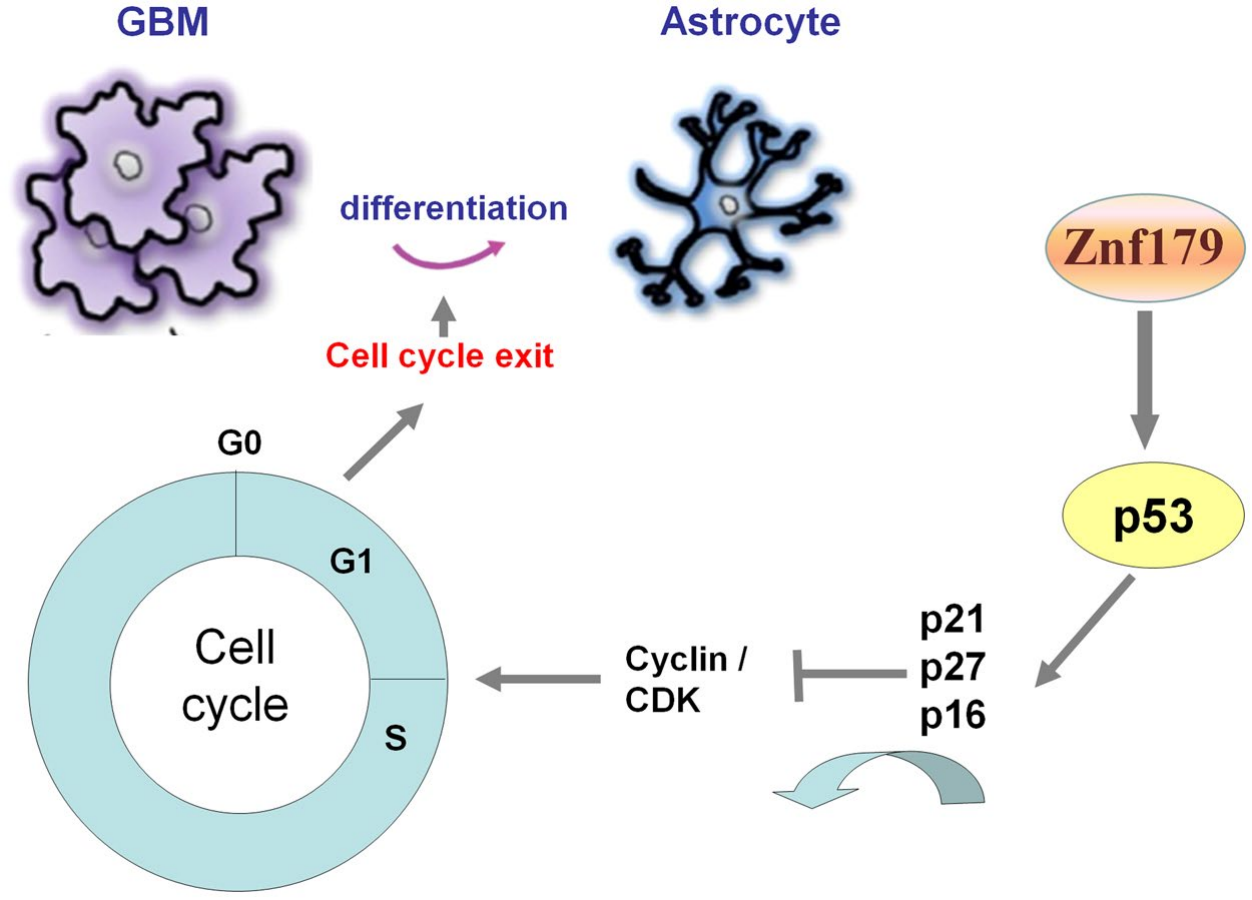
Summary diagram of the novel role of Znf179 in glioblastoma multiforme (GBM) cell cycle regulation and differentiation. Overexpression of Znf179 induces differentiation of primary GBM cells accompanied by elevated glial fibrillary acidic protein (GFAP) expression through upregulating several cell-cycle-related factors, i.e., p53, p21, and p27, and inducing cell-cycle arrest in the G_0_/G_1_ phase.

Until today, there is still no effective therapeutic treatment for the high-grade astrocytoma GBM, and relatively little is known about the real causes of gliomagenesis. Hence, the combinations of genetic manipulation of animal studies and bioinformatic database analyses are required to identify cellular and molecular mechanisms in glioma formation. Deregulation of differentiation and proliferation of neural stem cells (NSCs) into astrocytes during brain development and gliogenesis might contribute to glioma formation. Herein, we found that overexpression of Znf179 promotes neuronal differentiation of GBM cells and modulates the cell cycle through the p53-p21-p27 signaling pathway. Our genetic analysis showed that expression levels of Znf179 genes are highly correlated with prognosis and survival rates of patients with different glioma grades, further supporting that Znf179 may serve as a potential prognostic biomarker to allow the prediction of therapeutic prognosis and survival rates of glioma patients. Additional detailed molecular mechanisms will be needed for better therapeutic applications in the treatment of glioma patients. In this work, we suggested that Znf179 plays a role as a tumor-suppressor in human GBM through arresting the cell cycle in the G_1_ phase and subsequently inducing GBM differentiation. In conclusion, our data revealed that Znf179 can alter the phenotype of human primary GBM cells from an oncogenic state into a more-differentiated and less-oncogenic state (Fig. 6). Therefore, the anticancer effects of Znf179 and its molecular mechanism through p53-mediated cell-cycle regulation might be a potential target axis for the development of new therapeutic strategy in human GBM.

## Methods

### Cell culture and transfection

Animals used in this study were performed according to the guidelines of the Taipei Medical University (TMU) Institutional Animal Care and Use Committee (IACUC). All animals were housed in an air-conditioned vivarium with free access to food and water and a 12/12-h light/dark cycle. The primary cerebellar granule neurons were prepared from 7-day-old wild-type or Znf179-knockout B6 mice. The cerebellum was mechanically dissociated and filtered through a 70-mm nylon mesh filter (Small Parts, Miami Lakes, FL, USA). Cells were plated onto a plastic culture plate that was pre-coated with poly-L-lysine (30 mg/ml; Sigma-Aldrich, MO, USA) and maintained in Dulbecco’s modified Eagle’s medium (DMEM) Glutamax-I (Invitrogen, CA, USA) medium containing 10% fetal bovine serum (FBS; Invitrogen), 20 mM KCl, and 100 U/ml penicillin and streptomycin (Invitrogen), and completed with B27 supplements (Invitrogen). The growth of non-neuronal cells was inhibited by the addition of the antimitotics, uridine, and fluorodeoxyuridine (10 mM; Sigma-Aldrich), for 18 h after seeding. Cells were kept at 37 °C in a humidified atmosphere of 5% CO_2_ and 95% air. All of the methods were carried out in the accordance of the protocols approved by the TMU Institutional Animal Care and Use Committee (IACUC) under permit number LAC-2013-0120 and LAC-2015-0330. Neuro 2a (N2a) cells were cultured in Eagle’s minimum essential medium (MEM; Invitrogen), supplemented with 10% (vol/vol) FBS and 1% penicillin-streptomycin (Invitrogen). N2a cells were induced to differentiate in MEM medium with 2% serum. DNA transfection of N2a or GBM cells was carried out with Lipofectamine 2000 (Invitrogen) according to the manufacturer’s instructions.

### Tumor specimens and primary tumor cultures

Following informed consent and in accordance with the appropriate institutional review boards, GBM samples were obtained from a patient undergoing surgery at the Taipei Medical University Hospital (Taipei, Taiwan). Tumors were classified as GBM based on WHO criteria after examination by pathologists. Within 1~3 h of surgical resection, the tumor mass was mechanically and enzymatically dissociated into single cells. GBM cells were cultured in DMEM (Invitrogen) with 10% FBS (Invitrogen). To induce differentiation of GBM cells, transfected GBM cells were seeded in DMEM/F12 (Invitrogen), B27 supplements (Invitrogen), human recombinant basic fibroblast growth factor (bFGF), and epidermal growth factor (EGF) (50 ng/ml each; Invitrogen), and 1% FBS (Invitrogen)^20^. All of the protocols has been approved by expedited review process of the TMU-Joint Institutional Review Board (IRB) on June 30, 2010, valid June 30, 2011, and accepts the monitoring of IRB under approval number 20100611.

### SAGE Anatomic Viewer

The public SAGE Anatomic Viewer website (https://cgap.nci.nih.gov/SAGE/AnatomicViewer) provides highly intuitive, visual displays of human and mouse gene expression, based on a unique analytical process^21^ that reliably matches SAGE tags, 10 or 17 nucleotides in length, to known genes. It displays gene expression in human normal and malignant tissues by shading each organ in one of ten colors, each representing a different level of gene expression. Gene expression levels are based on the analysis of counts of SAGE tags, which are either “short” (10 bp), including “extracted short” (10 bp extracted from 17 bp tag), or “long” (17 bp). Expression levels are color-coded using the same color scheme as the SAGE Anatomic Viewer.

### Flow cytometric analysis

Cells were trypsinized, collected by centrifugation, resuspended, and washed in phosphate-buffered saline (PBS) before fixing in 75% ethanol for 1 h at room temperature. Fixed cells were incubated with 20 mg/ml propidium iodide, 10 mg/ml RNase A, and 0.1% Triton X-100 for 30 min before analysis. The cell cycle was analyzed using a BD FACS Calibur flow cytometer (BD Bioscience, Franklin Lakes, NJ, USA), and data were analyzed using WinMDI software (Joseph Trotter, Scripps Research Institute, La Jolla, CA, USA).

### Western blot analysis

Cells were lysed in modified radioimmunoprecipitation assay (mRIPA) buffer (150 mM NaCl, 50 mM Tris-HCl (pH 7.8), 0.1% NP-40, and 0.5% Triton X-100) with the addition of a protease inhibitor cocktail (Roche, Basel, Switzerland). Cell debris was removed by centrifugation at 12,000 rpm for 10 min at 4 °C, and the supernatant was used for a Western blot analysis. Protein concentrations were determined by a Bio-Rad dye-binding method (Bio-Rad, Hercules, California, USA) with bovine serum albumin (BSA) as the standard. Equal amounts of sample were separated by 10% sodium dodecylsulfate polyacrylamide gel electrophoresis (SDS-PAGE). The resolved proteins were then electroblotted onto polyvinylidene difluoride (PVDF) membranes (Millipore, Temecula, California, USA). Membranes were blocked with 5% nonfat milk and then sequentially incubated with primary and secondary antibodies. After washing, blots were processed for visualization using an enhanced chemiluminescence system (Millipore). Blots were then exposed to x-ray film to obtain fluorographic images. The following primary antibodies were used: rat polyclonal anti-GFAP (Invitrogen), mouse monoclonal anti-Tuj1 (1:10^4^, Promega, Madison, WI, USA), mouse monoclonal anti-p53 (1:3000, Cell Signaling, Danvers, MA, USA), rabbit polyclonal anti-nestin (1:1000, Santa Cruz Biotechnology, Santa Cruz, CA, USA), rabbit polyclonal anti-CD133 (1:1000, Proteintech, Rosemont, IL, USA), mouse monoclonal anti-p21 (1:500, BD Bioscience), rabbit polyclonal anti-p27 (1:500, Santa Cruz Biotechnology), rabbit polyclonal anti-pFOXO3 (1:1000, Cell Signaling), rabbit polyclonal anti-EGFR (1:1000, Cell Signaling), rabbit polyclonal anti-CDK4 (1:1000, Cell Signaling), mouse monoclonal anti-GAPDH (1:1000, Genetex, Irvine, CA, USA), and rabbit polyclonal anti-Znf179 (1:2000, generated in house).

### Immunofluorescent assay and analysis

Cells were fixed with 4% ice-cold paraformaldehyde at 4 °C for 20 min and then permeabilized with PBS-0.1% Triton X-100 for 7 min at room temperature. After blocking with 10% donkey serum for 1 h at room temperature, cells were incubated overnight at 4 °C with specific primary antibodies. The primary antibodies used for immunofluorescent were: rabbit polyclonal anti-MAP2 (1:200, Millipore), rat polyclonal anti-GFAP (1:1000, Invitrogene), mouse monoclonal anti-Tuj1 (1:500, Promega) and mouse monoclonal anti-p53 (1:200, Cell Signaling). After three time washes with PBS, cells were then incubated at room temperature for 1.5 h with DAPI (Invitrogen), Alexa Fluor 488-conjugated goat anti-rabbit, Alexa Fluor 488-conjugated goat anti-mouse immunoglobulin G (IgG) secondary antibody Alexa Fluora 546-conjugated goat anti-rabbit, or Alexa Fluor 546-conjugated goat anti-mouse IgG secondary antibody (Invitrogen). The coverslips were then mounted with fluorescent mounting solution and observed by confocal microscopy. Images were examined using a Leica DM6000B fluorescence microscope and quantified by MetaMorph software (Molecular Devices, Downingtown, PA, USA). For quantitative analyses of differentiated cells in Fig. 1, images from at least five representative fields per sample were taken and analyzed by MetaMorph software. In Fig. 1A and B, MAP2(+) cells represent granule cells with significant neurite branching and dendritic arborization. MAP2(+) and total cells were counted in at least five fields of view (FOVs), and at least 300 cells were counted in total by MetaMorph. Also, in Fig. 1C and D, significant neurite branching was observed in differentiated N2a cells. Total and differentiated N2a cells were determined in at least five FOVs, and at least 600 cells were counted in total by MetaMorph. Numbers of individual cells were calculated with the Integrated Morphometry Analysis of Metamorph. Percentages of MAP2(+) or differentiated N2a cells were calculated using the following formula: total number of MAP2(+) or differentiated N2a cells/total cells.

### Immunohistochemistry (IHC)

The brain tumor tissue microarray containing 42 malignant grade IV GBM tissue samples and 27 normal tissue samples was purchased from US Biomax. IHC staining was performed on 5-µm-thick tissue sections using an automated immunostainer (Ventana Discovery XT autostainer, ST?, USA). Briefly, sections were first dewaxed with xylene followed by rehydration in graded alcohol. Antigens were retrieved by heat-induced antigen retrieval for 10 min with citrate buffer. Slides were reacted with a polyclonal Znf179 antibody (1:500; Novus, city?, ST?, USA). After reaction with a linked antibody, sections were developed with diaminobenzidine (DAB), counterstained with hematoxylin, dehydrated, and mounted. IHC staining was assessed by a pathologist (CLC). Znf179 IHC expression was scored based on a four-tiered approach for cytoplasmic Znf179 IHC expression in tumor cells: negative staining (0), weak staining (1), moderate staining (2), and strong staining (3). Negative staining and weak staining were defined as low Znf179 IHC expression, and moderate to strong staining was defined as high Znf179 IHC expression.

### Measurement of numbers and sizes of glioma tumor spheres

Primary GBM cells were differentiated in DMEM/F12 medium containing B27, bFGF (50 ng/ml), EGF (50 ng/ml), and 1% FBS. Numbers of spheres were calculated with the Integrated Morphometry Analysis of Metamorph. The diameter of each glioma tumor sphere was also measured by Metamorph. Average numbers of glioma tumor spheres and sphere sizes of GBM cells were determined in at least three independent experiments.

### Statistical analysis

All experiments were conducted at least in triplicate, and results are expressed as the mean ± standard error of the mean (SEM). Statistical analyses were conducted using one-way analysis of variance (ANOVA) or Student’s *t*-test.

## Electronic supplementary material

Supplementary figures and legends

## References

[1] Beier D, Schulz JB, Beier CP (2011). Chemoresistance of glioblastoma cancer stem cells–much more complex than expected. Mol Cancer.

[2] Meier B (2001). The first patient to undergo coronary angioplasty–23-year follow-up. N Engl J Med.

[3] Mischel PS, Nelson SF, Cloughesy TF (2003). Molecular analysis of glioblastoma: pathway profiling and its implications for patient therapy. Cancer Biol Ther.

[4] Louis DN (2007). The 2007 WHO classification of tumours of the central nervous system. Acta Neuropathol.

[5] Uchida N (2000). Direct isolation of human central nervous system stem cells. Proc Natl Acad Sci USA.

[6] Bao S (2006). Glioma stem cells promote radioresistance by preferential activation of the DNA damage response. Nature.

[7] Korur S (2009). GSK3beta regulates differentiation and growth arrest in glioblastoma. PLoS One.

[8] Aguado T (2007). Cannabinoids induce glioma stem-like cell differentiation and inhibit gliomagenesis. J Biol Chem.

[9] Kang TW (2014). Growth arrest and forced differentiation of human primary glioblastoma multiforme by a novel small molecule. Sci Rep.

[10] Pao PC (2011). A novel RING finger protein, Znf179, modulates cell cycle exit and neuronal differentiation of P19 embryonal carcinoma cells. Cell Death Differ.

[11] Inoue S (1997). A novel RING finger protein, BFP, predominantly expressed in the brain. Biochem Biophys Res Commun.

[12] Orimo A (1998). Molecular cloning, localization, and developmental expression of mouse brain finger protein (Bfp)/ZNF179: distribution of bfp mRNA partially coincides with the affected areas of Smith-Magenis syndrome. Genomics.

[13] Swartling FJ, Cancer M, Frantz A, Weishaupt H, Persson AI (2015). Deregulated proliferation and differentiation in brain tumors. Cell Tissue Res.

[14] Chen J, McKay RM, Parada LF (2012). Malignant glioma: lessons from genomics, mouse models, and stem cells. Cell.

[15] Hulleman E, Helin K (2005). Molecular mechanisms in gliomagenesis. Adv Cancer Res.

[16] Mischel PS, Cloughesy TF, Nelson SF (2004). DNA-microarray analysis of brain cancer: molecular classification for therapy. Nat Rev Neurosci.

[17] Mizuno H, Kitada K, Nakai K, Sarai A (2009). PrognoScan: a new database for meta-analysis of the prognostic value of genes. BMC Med Genomics.

[18] Panieri E, Santoro MM (2016). ROS homeostasis and metabolism: a dangerous liason in cancer cells. Cell Death Dis.

[19] Su TC (2016). The sigma-1 receptor-zinc finger protein 179 pathway protects against hydrogen peroxide-induced cell injury. Neuropharmacology.

[20] Lee J (2006). Tumor stem cells derived from glioblastomas cultured in bFGF and EGF more closely mirror the phenotype and genotype of primary tumors than do serum-cultured cell lines. Cancer Cell.

[21] Boon K (2002). An anatomy of normal and malignant gene expression. Proc Natl Acad Sci USA.

