# Author Correction: Znf179 induces differentiation and growth arrest of human primary glioblastoma multiforme in a p53-dependent cell cycle pathway

**DOI:** 10.1038/s41598-018-30081-w

**Published:** 2018-09-03

**Authors:** Kuen-Haur Lee, Chi-Long Chen, Yi-Chao Lee, Tzu-Jen Kao, Kai-Yun Chen, Chih-Yeu Fang, Wen-Chang Chang, Yung-Hsaio Chiang, Chi-Chen Huang

**Affiliations:** 1 Graduate Institute of Cancer Biology and Drug Discovery, College of Medical Science and Technology, Taipei Medical University, Taipei, Taiwan; 2 Department of Pathology, Taipei Medical University Hospital, Taipei, Taiwan; 3 Department of Pathology, School of Medicine, College of Medicine, Taipei Medical University, Taipei, Taiwan; 4 Graduate Institute of Neural Regenerative Medicine, College of Medical Science and Technology, Taipei Medical University, Taipei, Taiwan; 5 Department of Pathology, Wan Fang Hospital, Taipei Medical University, Taipei, Taiwan; 6 Graduate Institute of Medical Sciences, College of Medicine, Taipei Medical University, Taipei, Taiwan

Correction to: *Scientific Reports* 10.1038/s41598-017-05305-0, published online 06 July 2017

In the original version of this Article, Chi-Long Chen and Chih-Yeu Fang were incorrectly listed as being affiliated with “Pathology Department, Taipei Medical University, Taipei, Taiwan”. The correct affiliations are listed below:

Chi-Long Chen

Department of Pathology, Taipei Medical University Hospital, Taipei, Taiwan

Department of Pathology, School of Medicine, College of Medicine, Taipei Medical University, Taipei, Taiwan

Chih-Yeu Fang

Department of Pathology, Wan Fang Hospital, Taipei Medical University, Taipei, Taiwan

This error has now been corrected in the PDF and HTML versions of this Article, and in the accompanying Supplementary figures and legends file.

