# Supplementary material for: Znf179 induces differentiation and growth arrest of human primary glioblastoma multiforme in a p53-dependent cell cycle pathway
Source: Sci Rep. 2017 Jul 6;7:4787. doi: 10.1038/s41598-017-05305-0 (PMC5500472; doi:10.1038/s41598-017-05305-0)
Supplement: Supplementary file 1 — Supplementary figures and legends [file 41598_2017_5305_MOESM1_ESM.docx]

Znf179 induces differentiation and growth arrest of human primary glioblastoma multiforme in a p53-dependent cell cycle pathway

Kuen-Haur Lee 1, #, Chi-Long Chen 2,3, #, Yi-Chao Lee 4, Tzu-Jen Kao 4, Kai-Yun Chen 4, Chih-Yeu Fang 5, Wen-Chang Chang 6, Yung-Hsaio Chiang 4, Chi-Chen Huang 4,*

1. Graduate Institute of Cancer Biology and Drug Discovery, College of Medical Science and Technology, Taipei Medical University, Taipei, Taiwan

2. Department of Pathology, Taipei Medical University Hospital, Taipei, Taiwan

3. Department of Pathology, School of Medicine, College of Medicine, Taipei Medical University, Taipei, Taiwan

4. Graduate Institute of Neural Regenerative Medicine, College of Medical Science and Technology, Taipei Medical University, Taipei, Taiwan

5. Department of Pathology, Wan Fang Hospital, Taipei Medical University, Taipei, Taiwan

6. Graduate Institute of Medical Sciences, College of Medicine, Taipei Medical University, Taipei, Taiwan

# Co-Authors

* Corresponding Author

Please send all correspondence to: Chi-Chen Huang

E-mail: hcc0609@tmu.edu.tw

Supplementary Figure S1. Neural stem/progenitor cell markers, CD133 and nestin,

were equivalent among Znf179-overexpressing or non-overexpressing GBM cells. (A,

B) Primary GBM-derived cells stably expressing GFP-Znf79 were differentiated and

immunostained with an anti-pnestin antibody. Quantification of the percentage of

nestin(+) cells is shown in the histogram. (groups were compared by a t-test,

two-tailed p values). Scale bar: 50 μm. (C) Total cell lysates of N2a cells were

analyzed by Western blotting using anti-nestin and anti-β actin antibodies.

Quantification analysis of the expression levels of nestin is shown in the histogram.

(groups were compared by a t-test, two-tailed p values).

Supplementary Figure S2.Uncropped images of western blot analysis for Figure 1E.

Supplementary Figure S3.Uncropped images of western blot analysis for Figure 3D.

Supplementary Figure S4. Uncropped images of western blot analysis for Figure 4A

(A) and Figure 4B (B).

**(A)**

**Fig. S1**

**Control Nestin**

**DAPI**

**Flag-Znf179**

**Nestin**

**DAPI**

**5oum**

**GFP**

**Nestin**

**GFP-Znf179**

**Nestin**

***DAPI***

**(C)**

Flag-Znf179 GFP-Znf179

- +

- +

**33.6%**

**30.2%**

**28.6%**

**Nestin B-actin**

Nestin (+) / DAPI cells (%)

go

0.5

**GFP**

*90/*

**Flag**

**GFP**

**Falg-179**

**Flag**

**GFP-179**

**GFP-179**

Flag-znf179

**Fig. S2**

**Serum GFP-Znf179**

**N2a 10% 2% - + - +**

**Serum GFP-Znf179**

**N2a 10% - +**

**2% -**

**+**

**-**

**Znf179**

**MAP2**

-

---

**GFAP GFAP**

Ae

8

**-**

**q-tubulin**

**Znf179**

Bil

-

-

-Tuj-1

.

----

-GAPDH

**GFP-Znf179**

**Flag-Znf179**

. +

Fig. S3

**Nestin**

**CD133**

o sa a

**Znf179**

**GFAP**

48

**Znf179**

**GFAP**

**B-actin**

Fig. 54

**Flag - Znf179**

**Flag-Znf179**

-

+

-

+

-

+

-

+

-

+

-

+

**p53**

**Flag-Znf179**

**GAPDH GAPDH**

Flag - Znf179

+ -

+

**EGFR**

**Foxo3**

Cyclin D1 p27

**Znf179**

25

**cdk4**

**p21**

**S**

**.**

**O**

**GAPDH GAPDH**

**GAPDH**
